# Transgenes of the Mouse Immunoglobulin Heavy Chain Locus, Lacking Distal Elements in the 3′ Regulatory Region, Are Impaired for Class Switch Recombination

**DOI:** 10.1371/journal.pone.0055842

**Published:** 2013-02-08

**Authors:** Wesley A. Dunnick, Jian Shi, Clinton Fontaine, John T. Collins

**Affiliations:** Department of Microbiology and Immunology, University of Michigan Medical School, Ann Arbor, Michigan, United States of America; St. Jude Children’s Research Hospital, United States of America

## Abstract

The immunoglobulin heavy (H) chain class switch is mediated by a deletional recombination event between µ and γ, α, or ε constant region genes. This recombination event is upregulated during immune responses by a regulatory region that lies 3′ of the constant region genes. We study switch recombination using a transgene of the entire murine H chain constant region locus. We isolated two lines of mice in which the H chain transgenes were truncated at their 3′ ends. The truncation in both transgenic lines results in deletion of the 3′-most enhancer (HS4) and a region with insulator-like structure and activities. Even though both truncated transgenes express the µ H chain gene well, they undergo very low or undetectable switch recombination to transgenic γ and α constant region genes. For both transgenic lines, germline transcription of some H chain constant regions genes is severely impaired. However, the germline transcription of the γ1 and γ2a genes is at wild type levels for the transgenic line with the larger truncation, but at reduced levels for the transgenic line with the smaller truncation. The dramatic reduction in class switch recombination for all H chain genes and the varied reduction in germline transcription for some H chain genes could be caused by (i) insertion site effects or (ii) deletion of enhancer elements for class switch recombination and transcription, or (iii) a combination of both effects.

## Introduction

During an antigen-driven immune response, B cells can change their expression from IgM to IgG, IgA, or IgE, which is due to a change from µ to γ, α, or ε H chain expression. The H chain class switch is mediated by a deletion event that begins in the intron between the variable (V) region coding exon and the µ constant (C) region coding exons and ends in switch regions that are 1–10 kb in length, and lie upstream of the γ, α, or ε coding exons [1]. The process is referred to as class switch recombination (CSR) to emphasize the recombination event between the µ and γ, α, or ε genes that exchanges one H chain C region for another.

**Figure 1:**
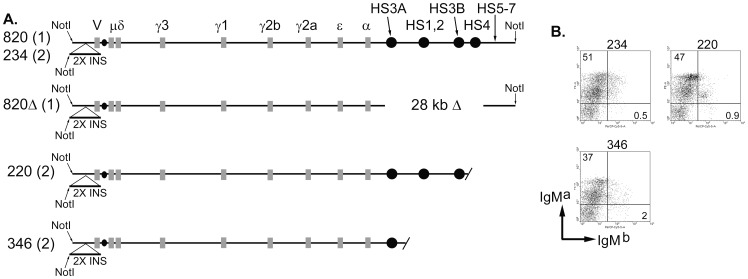
Structure and expression of truncated H chain transgenes. (A) Schematics of H chain transgenes. The name of the transgenic line, with copy number in parentheses, is shown to the left of each schematic. Coding exons are depicted as grey rectangles, and enhancers are black circles. An insertion of two copies of the chicken β-globin insulator [24] 3 kb 5′ of VDJ exon is indicated (“2X INS”). A *Not*I restriction site was engineered at the 5′ end of this insertion, and this *Not*I site is the 5′ end of the transgenic insertion; the 11 kb *Not*I fragment at the 5′ end of the BAC is not included in the fragment purified for oocyte injection. (B) Transgenic surface IgM^a^ expression. Transgenic line numbers are shown above the data. Percentages of total lymphocytes in the upper left and lower right quadrants are indicated. Mean fluorescence intensity of transgenic surface IgM^a^ expression: line #234∶66, line #220∶89, and line #346∶45. These results are representative of three or more independent experiments for each transgenic line.

CSR is silent in resting B cells, but must be dramatically upregulated during antigenic stimulation, with help from T cells. A regulatory region is located 3′ of the Cα gene and includes four enhancer segments, called HS3A, HS1,2, HS3B, and HS4, (Fig. 1A). We refer to the four enhancers collectively as the 3′ enhancers. These segments were identified as DNase I hypersensitivity sites (HS) and encode B cell-specific transcriptional enhancers [2]–[11]. Consistent with their synergy in transcriptional enhancement, deletion of any single 3′ enhancer has little effect on CSR [12]–[14]. However, the 3′ enhancers were shown to be important for upregulation of CSR, in that insertion mutations or deletion of two of them leads to a reduction in CSR to most H chain genes [15]–[17]. Although the endogenous 3′ enhancer region has been difficult to target using homologous recombination in ES cells, a 28–30 kb deletion of all four 3′ enhancers was shown to essentially eliminate CSR to all α, ε, and all four γ genes using a transgenic system [18] or targeting of the endogenous locus [19]. Deletion of two or three elements by 1.1 to 1.3 kb deletions has an intermediate effect on CSR, and deletion of all four elements by the smaller deletions essentially eliminates CSR [17], [19]–[21]. Thus, it appears that most of the upregulation of CSR is controlled by the four 3′ enhancers.

Three additional DNase I hypersensitive sites have been identified 3′ of HS4 and are called HS5, HS6, and HS7 [22] (see also Fig. 1A). These DNase I HS have minimal transcriptional enhancer activity. However, this region is rich in binding sites for the CCCTC binding factor (CTCF) [22], [23]. CTCF binding sites are well-correlated with insulators [24], and the HS5-7 region has some activities of an insulator element [22]. Deletion of HS5-7 from the endogenous locus has some effects on H chain gene expression, but the effects tend to be small [25]. We have studied CSR by using a 230-kb transgene that includes an assembled VDJH2 exon, all eight constant region genes, the four 3′ enhancers, and an additional 15 kb of DNA that includes HS5, HS6, and HS7 (Fig. 1A). We identified two founders that had truncations of the H chain transgene at its 3′ end, retaining all CH genes, but suffering deletions of HS4 (or HS4, HS3B, and HS1,2) and the 15 kb including HS5–7. We characterized transgenic µ H chain expression and CSR in mice carrying the truncated H chain loci.

## Materials and Methods

### Ethics Statement

All work with mice was approved by the University of Michigan Committee on Use and Care of Animals (protocol 08147), and was conducted in accordance with that protocol.

### Transgenic Mice and Cell Culture

Fertilized eggs were injected with the 230-kb insert of a BAC containing the H chain constant region locus [21], [26]. The specific mice analyzed in this study were originally identified as founder mice that were positive for the transgenic VDJ, but negative for transgenic HS4. These founder mice were further characterized for gene content of the H chain locus, and the two founders (#220 and #346) were found to retain all of the H chain constant region genes and HS3A. A third truncated line (#757) was found to lack two or more transgenic CH genes, and so was eliminated from the study. The removal of the data for line #757 is indicated by grey lines in some figures. Transgenic lines were established by backcrosses to C57BL/6 (*Igh* ^b^). Splenocytes, depleted of T lymphocytes [27], were cultured in RPMI 1640 supplemented with 10% FBS, penicillin, streptomycin, glutamine, and 50 µM 2ME. For harvesting of RNA, cells were cultured for three days at 1.5 million per ml in 5–10 ml cultures. LPS (25 µg/ml; Sigma L7261), CD40 ligand (CD40L)-expressing Sf9 cells (150,000/ml–RNA cultures–or 20,000 per ml–Ig secretion cultures [28]), IL-4 (35 ng/ml; Biosource PMC0045), interferon-γ (10 ng/ml; Biosource PMC4034), and human TGF-β (4 ng/ml; Preprotech 100-21C) were added to the cultures in various combinations. For analysis of secreted immunoglobulins, cells were cultured for seven days at 100,000 per ml in 1-ml cultures.

### Analysis of Cell Surface Ig Expression, RNA Expression, and Ig Secretion

Cell surface expression was analyzed using anti-IgM^a^, anti-IgM^b^, anti-B220, and anti-IgD antibodies (all from BD Biosciences), with data collection, using a lymphocyte gate, on a FACS Canto. The H chain transgene was derived from strain 129 mice (*Igh* ^a^), while the endogenous (C57Bl/6) genes are Igh^b^. Thus, cell surface expression of transgenic IgM is detected with anti-IgM^a^ and cell surface expression of endogenous IgM is detected with anti-IgM^b^. We could distinguish endogenous and transgenic DNA or cDNA by multiple sequence polymorphisms between the two H chain alleles. Analysis of germline transcripts included amplification of both transgenic and endogenous germline transcripts with gene-specific I exon and CH primers, followed by restriction enzyme digestion [21]. (See Table S1 for primer sequences, restriction digests, and predicted fragment sizes). Analysis of post-switch transcripts included amplification using an Iµ primer or a primer specific for the transgenic VDJ and CH-specific primers [26] (see Table S1 for primer sequences). All RT-PCR reactions included ^32^P-dATP in the reaction mixture. PCR products, or restriction fragments derived from PCR products, were quantified on a PhosphorImager, with background subtraction of an area in the same lane lacking any radioactive fragments. ELISA for secreted transgenic IgG1 included capture with anti-IgG1^a^ (BD Biosciences) and development with AP-conjugated anti-IgG1 (Southern Biotech). The transgene has a Flag tag inserted into the CH3 exon of the Cγ2a gene (18). ELISA with plates coated with anti-Flag (Sigma F1804) and development with anti-IgG2a (Southern Biotech) detected transgenic IgG2a.

### Digestion-Circularization-PCR (DC-PCR)

DNA was prepared from 3-day cultures of transgenic B cells, digested with *Eco*RI, and ligated at high dilution to form circles [29]. Primers in the 5′ end of the *Eco*RI fragment containing Sµ and the 3′ end of the *Eco*RI fragment containing Sγ1 or Sγ2a were used to amplify DC-PCR products in the presence of ^32^P-dATP [18]. DC-PCR products were purified by 6% PAGE gels and digested with *Mbo*I or *Dde*I. See Table S2 for PCR primers and predicted fragment sizes. The resulting restriction fragments were fractionated by 8% PAGE.

## Results

### Identification of Mice with Truncated H Chain Transgenes

**Figure 2:**
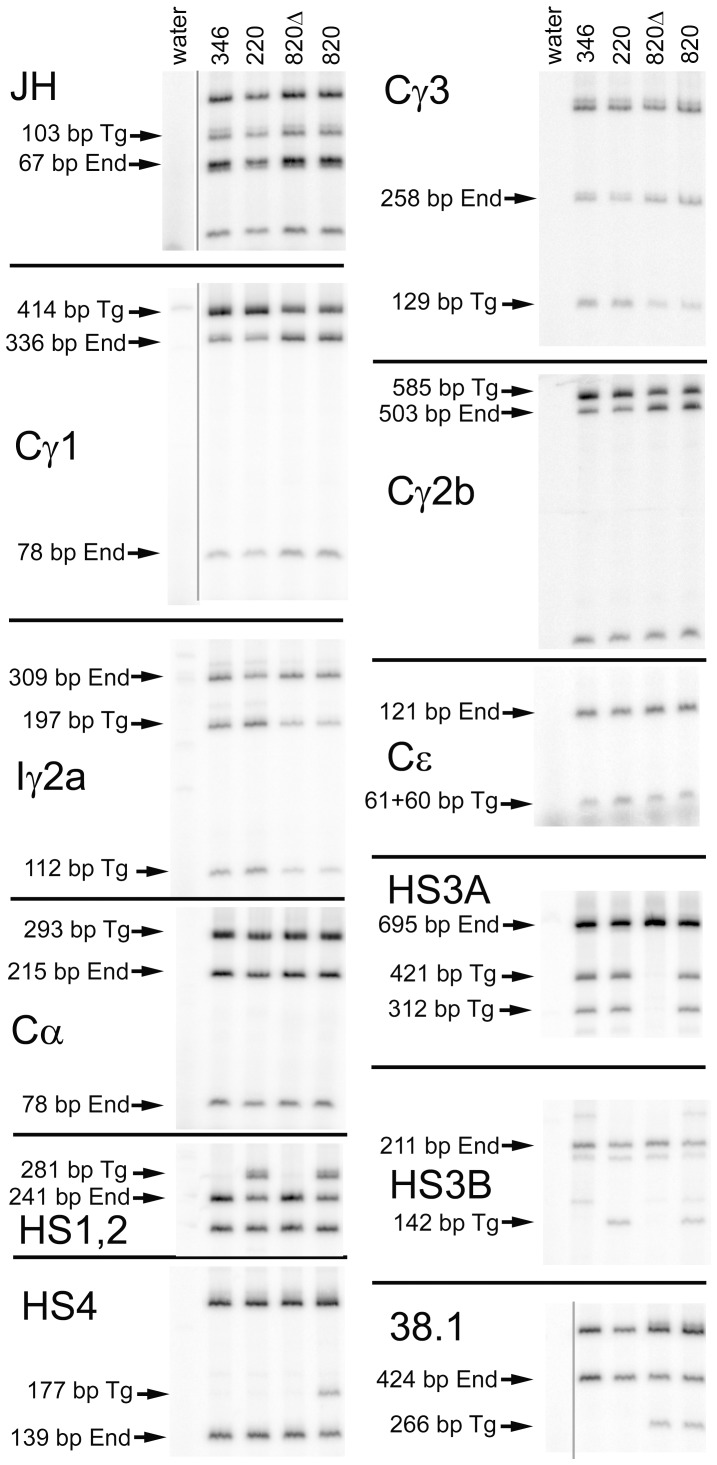
Gene composition of transgenic lines. Tail DNA for the transgenic lines indicated above the lanes were amplified for the gene segment indicated to the left of each set of PCR results. After amplification in the presence of ^32^P-dATP, the products were digested with a restriction enzyme. Restriction fragments that arise from the transgene only (Tg) or the endogenous genes only (End) are indicated. See Table S3 for PCR primers, restriction digests, and predicted fragment sizes. The designation of these fragments are consistent with the DNA sequences of the relevant C57BL/6 or strain 129 DNA, and have been confirmed by comparison to C57BL/6 DNA [18]. Other restriction fragments are shared by both the transgene and endogenous genes.

While screening transgenic founders with immunoglobulin H chain genes, we identified founder #220, that retained HS3A; HS1,2; and HS3B, but was truncated at the 3′ end of the transgene, and so lacked HS4 and the 15 kb of DNA 3′ to HS4. This 15 kb includes three additional B-cell specific DNase I hypersensitive sites (HS5, HS6, and HS7) [22]. A second founder (#346), from the same injection, retained HS3A only, lacking the other three 3′ enhancers and 15 kb downstream (Fig. 1A). Transgenic lines were established by breeding to C57BL/6 mice. Using surface IgM as a criterion, both line #346 and #220 express the transgene well (Fig. 1B). Both lines exhibited strong exclusion of the endogenous H chain genes, in that more than 94% of the B cells express transgenic IgM^a^, not endogenous IgM^b^. Relative to line #234, with a wild type H chain transgene, there was a small reduction in the percent of B cells expressing IgM^a^, and a small increase in the percent of B cells expressing IgM^b^ (Fig. 1B). We tested genomic DNA of lines #346 and #220, along with line #820 and #820Δ. Line #820 is an intact H chain transgene and so is a positive control for the seven H chain gene segments tested, HS1-4, and “38.1″. The sequence designated “38.1″ [22] is located 10 kb 3′ of HS4, but 5 kb 5′ of the end of the transgene. We have deleted HS1-4 from line #820Δ, but it retains all the C region genes and the 38.1 sequences [18]. These results verified that line #346 retained all H chain genes and HS3A in the transgene, and that line #220 retained all H chain genes, HS3A, HS1,2, and HS3B (Fig. 2). By digestion of the PCR products with a restriction enzyme that differentiates the transgene and the endogenous genes, we could compare the copy number of the transgene to that of the endogenous genes. In lines #346 and #220 the transgenic signal for the H chain genes was similar to that of the endogenous genes, indicating a copy number of two. Also, relative to the signal from endogenous genes in various transgenic lines, the signal in lines #346 and #220 was about twice that of line #820, a single copy transgenic line (Fig. 2).

### Secretion of IgG by B Cells with Truncated Transgenes

**Figure 3:**
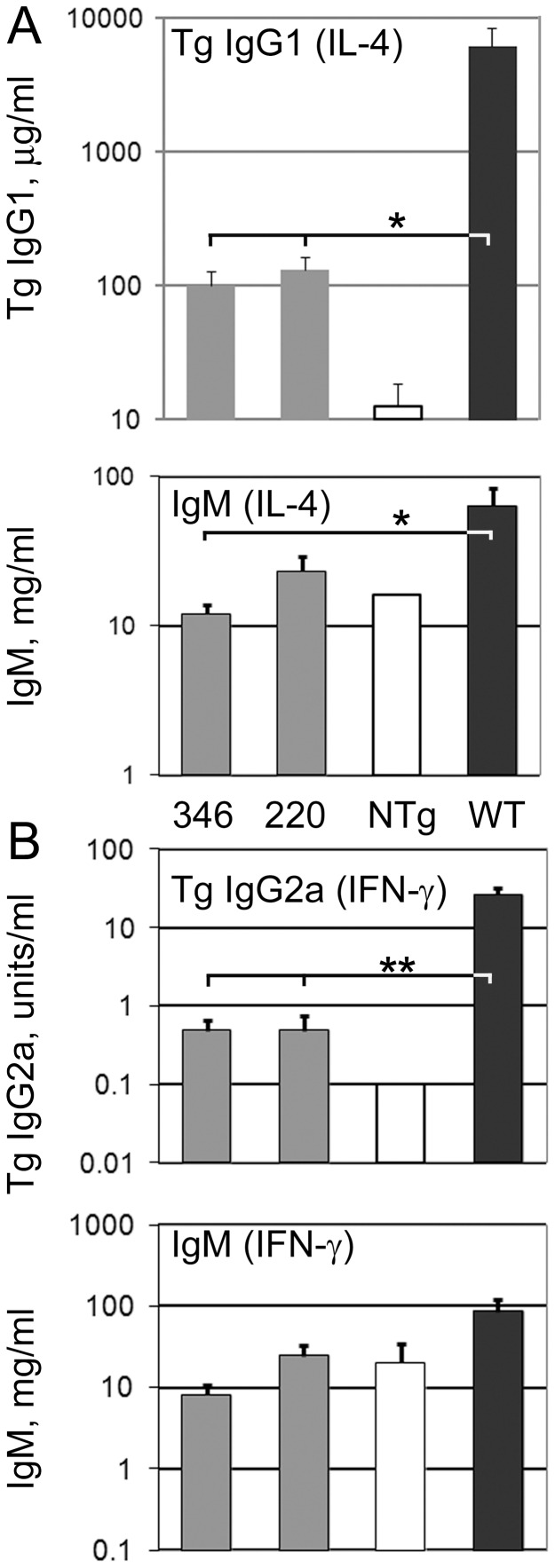
Expression of secreted IgG from the transgene. Data are presented as geometric means, with SE bars, of 5–14 independent experiments from transgenic mice or 1–3 experiments from nontransgenic (“NTg”) controls. Grey bars: lines #346 or #220; open bars: nontransgenic; black bars: wild type transgenic (lines #820 or #234). (A) Secretion of transgenic IgG1^a^ (upper) or total IgM (lower) from LPS+IL-4 or CD40L+IL-4 cultures. One culture from nontransgenic mice was tested for total IgM expression. (B) Secretion of transgenic (Flag-tagged) IgG2a (upper) or total IgM (lower) from LPS+interferon-γ or CD40L+interferon-γ cultures. No error bar is shown for nontransgenic IgG2a secretion, as all three determinations yielded undetectable Flagged-tagged IgG2a, designated 0.1 units per ml. Statistical significance (Students t test) is shown for comparison of line #346 or line #220 results to wild type (positive control) or nontransgenic (negative control) results, * 0.01<p<0.05 and **p<0.01. All other comparisons of #346 or #220 with wild type or nontransgenic results had p values >0.05.

We tested for CSR by the truncated H chain transgenes by measuring the amount of transgenic IgG secreted in cultures of transgenic B cells induced to undergo CSR. B cells with the truncated transgenes secreted an average of 101 or 131 µg/ml transgenic IgG1^a^ compared to an average of 6100 µg/ml secreted by wild type transgenes (Fig. 3A). This smaller amount of IgG1^a^ secretion was not significantly different than the background level defined by non-transgenic B cell cultures (Fig. 3A, p = 0.06 or greater). A random subset of the LPS+IL-4 or CD40L+IL-4 cultures was also tested for secretion of total IgM. Although all tested cultures included B lineage cells capable of IgM secretion, an average of 12 or 24 mg/ml of IgM secreted by the truncated H chain transgenes was reduced compared to 64 mg/ml from wild type transgenes (Fig. 3A, lower). Similar results were obtained for secretion of transgenic IgG2a in cultures with interferon-γ. Transgenic IgG2a secretion by B cells with the truncated transgenes was on average 0.5 units/ml compared to 27 units per ml by B cells with wild type transgenes (Fig. 3B). The secretion of IgG2a by B cells with the truncated transgenes was not statistically different than that of non-transgenic B cells. Total IgM secretion from B cells with the truncated transgenes cultured in LPS+interferon-γ or CD40L+interferon-γ (an average of 8 or 25 mg/ml IgM) was reduced compared to 85 mg/ml secreted from wild type transgenes. Thus, the expression of transgenic, secreted IgG was greatly reduced in B cells with the truncated heavy chain transgenes, even though expression of secreted IgM was only moderately reduced.

### Expression of Germline and Post-Switch Transcripts from Truncated Transgenes

**Figure 4:**
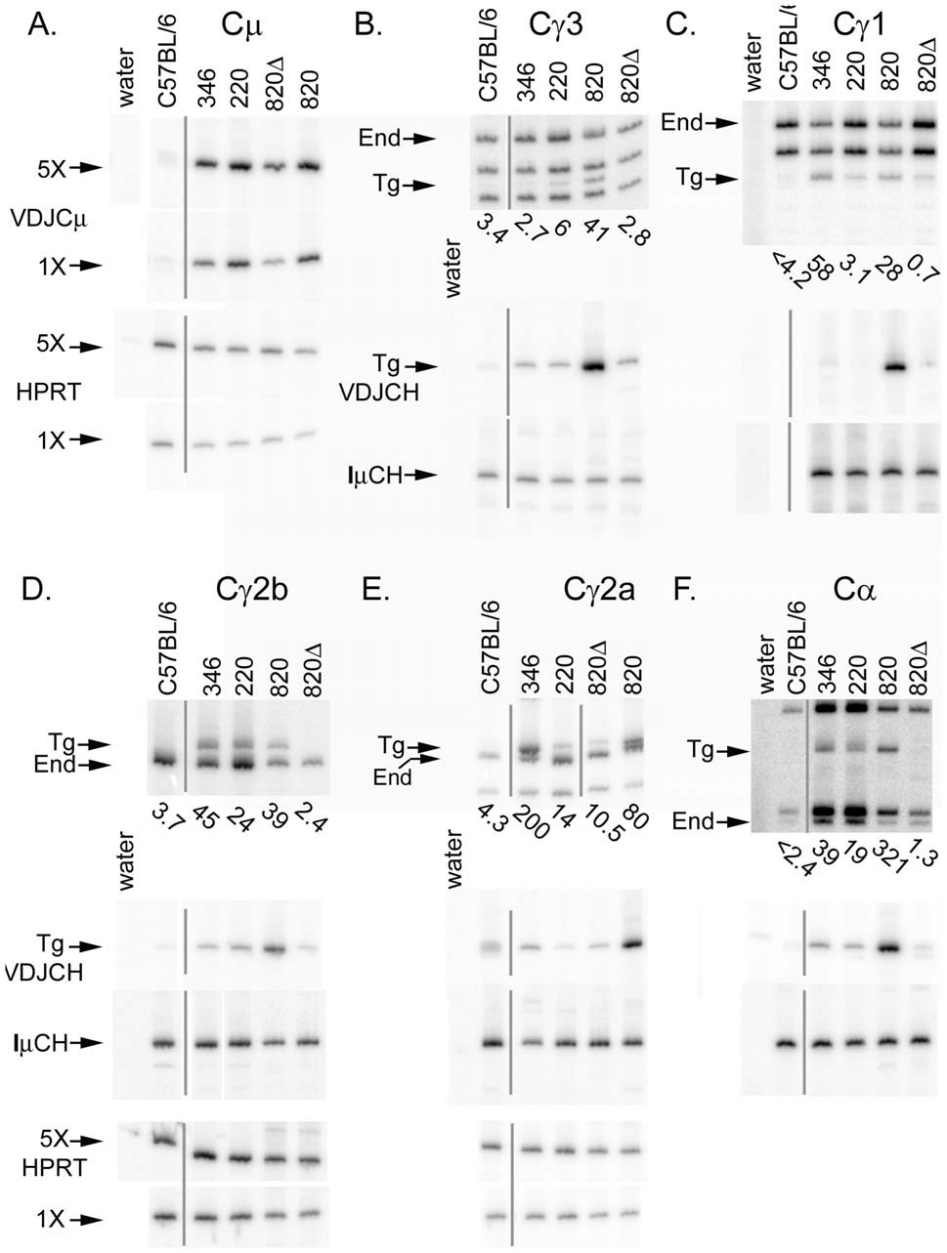
Expression of germline and post-switch transcripts from truncated H chain transgenes. B cells were cultured for three days, and RNA was prepared from the cultured cells. RNA expression was tested by semi-quantitative RT-PCR. (A) Expression of Cµ in RNA from B cells cultured with LPS; (B) Expression of Cγ3, (D) Expression of Cγ2b, and (F) Expression of Cα in RNA from B cells cultured in LPS+TGF-β; (C) Expression of Cγ1 in RNA from B cells cultured in LPS+IL-4; (E) Expression of Cγ2a in RNA from B cells cultured in CD40L+interferon-γ. For expression of germline transcripts (top set of panels for Cγ and Cα), RT-PCR products were digested with a restriction enzyme that cuts at polymorphisms between the transgene and endogenous genes. This allowed detection of germline transcripts from the transgene as well as germline transcripts from the endogenous genes. The portion of signal in the Tg fragments relative to the signal in the End fragments (x100) is shown below each lane. VDJCH transcripts (upper panel for Cµ and middle panels for Cγ and Cα) were amplified using a primer specific for the VD junction of the transgene. Total Iµ transcripts, from both the endogenous and transgene, were also detected (lower panels for Cγ and Cα). Example HPRT expression is shown for LPS (A), LPS+TGF-β (D), and CD40L+interferon-γ (E) RNA samples. The grey lines indicate that one lane was removed from the original PhosphorImager file. The results presented here are representative of multiple tests of germline transcripts, VDJCH transcripts, and IµCH transcripts from at least three independent cell cultures and RNA preparation for each transgenic line.

We tested germline transcription of these truncated transgenes by amplification of all germline transcripts, followed by digestion with restriction enzymes. The restriction enzyme used for each H chain gene was chosen to cut at a site that was polymorphic between the transgenes and endogenous genes. Hence, each restriction digest of the PCR products of germline transcripts yielded one or more fragments unique to the transgene and unique to the endogenous genes (Fig. 4). (Germline transcripts of the γ2a gene were distinguished, not by restriction enzyme digest, but by a migration polymorphism.) Since the RT-PCR products for germline transcripts derived from the endogenous genes and transgenes are identical except for 1–4 bp polymorphisms, they should be amplified with equal efficiency. Hence, the amount of RT-PCR products for germline transcripts from the endogenous genes acts as a normalization control for both the wild type and truncated H chain transgenes. We quantified the amount of germline transcripts by dividing the radioactivity in the transgene-specific bands by the amount of radioactivity in the endogenous gene-specific bands, and presented these values (multiplied times 100) below each lane in the upper panels of Fig. 4B to 4F. For example, cDNA from lines #346 and #220 yield little or no transgene-specific fragment of γ3 germline transcripts, even though abundant endogenous gene-specific fragments are detected (Fig. 4B, upper panel). Thus, the truncated H chain transgenes expressed small amounts of germline transcripts of the γ3 gene, similar to transgene that have a 28-kb deletion of the 3′ enhancer region (line #820Δ, Fig. 4B). Both truncated transgenes expressed abundant germline transcripts of the γ2b gene, a ratio of transgene to endogenous (x 100) of 24 or 45 from truncated transgenes compared to a ratio of 39 from wild type transgenes (Fig. 4D, upper panel). Expression of germline transcripts from the α gene was reduced to transgenic to endogenous ratios of 39 or 18 for the truncated transgenes compared to a ratio of 321for the wild type transgene (Fig. 4F, upper panel). B cells from line #346 expressed wild type levels of transgenic γ1 and γ2a transcripts, but line #220 expressed greatly reduced levels of germline transcripts of these two γ genes, similar to that of transgenes with a 28 kb deletion of the 3′ enhancer region (Fig. 4C and 4E, upper panels).

We estimated CSR by amplification of VDJCH transcripts, using a primer specific for the VD junction of the transgene. In general, these transcripts were not detected in RNA from nontransgenic (C57BL/6) B cells or detected as a faint, poorly defined, band. Consistent with μ expression on the cell surface, B cells from line #346 expressed slightly reduced levels of VDJCμ transcripts (relative to HPRT mRNA expression), and B cells from line #220 expressed similar levels of VDJCµ transcripts to the levels from B cells with an intact transgene (Fig. 4A). B cells from mice with an intact transgene (line #820) also expressed easily detectable VDJCγ or VDJCα transcripts, indicating good CSR. However, expression of all four types of VDJCγ and of VDJCα transcripts by B cells with the truncated transgenes was poor, as low as the expression from a transgene lacking the 3′ enhancer region (#820Δ, Fig. 4B, C, D, E, F). As an additional control for these experiments, we analyzed IµCH transcripts [30]. An IµCH RT-PCR detects CSR of the endogenous genes, which will undergo CSR even in the absence of an assembled VDJ exon. Since all samples, including those from truncated transgenes, expressed strong IµCγ or IµCα transcripts, the B cells in all samples were activated properly and received cytokine signals, yielded intact mRNA, etc.

**Figure 5:**
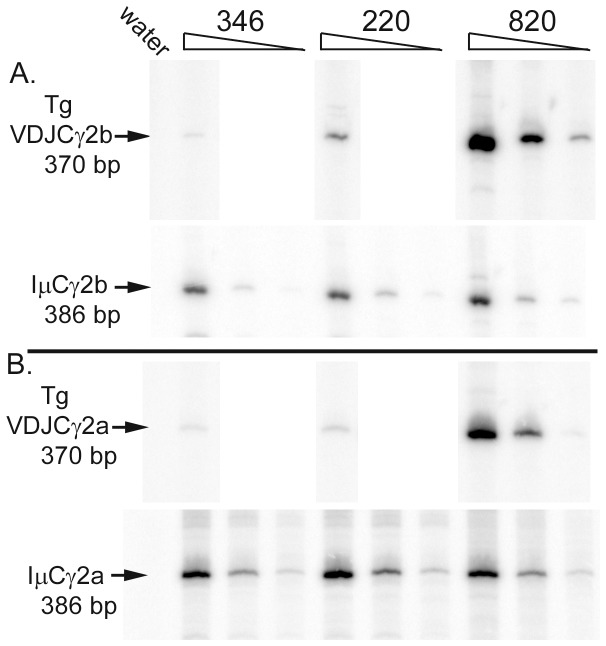
Semi-quantitative PCR evaluation of the levels of post-switch transcripts from truncated H chain transgenes. The samples tested in Figure 4, parts D and E, were re-evaluated after balancing for similar amount of IµCγ at three five-fold dilutions of cDNA (lower part of each panel). The same dilutions of cDNA were then tested for amounts of VDJCγb (A) or VDJCγ2a transcripts (B). RT-PCR products, with ^32^P-dATP incorporated, were visualized on a PhosphoImager.

To obtain better quantification of the amount of VDJCγ transcripts, we examined post-switch transcripts by semi-quantitative RT-PCR. We chose to examine VDJCγ2b and VDJCγ2a transcripts, as these samples yielded RT-PCR products that were apparently in greater quantity than those from H chain transgenes lacking all four 3′ enhancers (#820Δ, Fig. 4D, E). The samples were first normalized for total CSR, as estimated by IµCγ expression (Fig. 5). At most, the amounts of VDJCγ transcripts from truncated lines #346 and #220 were similar to those from a 1∶25 dilution of line #820, with an intact H chain transgene (Fig. 5). In some cases, the amount of VDJCγ transcripts from truncated lines #346 and #220 were less than those from a 1∶25 dilution of line #820.

**Figure 6:**
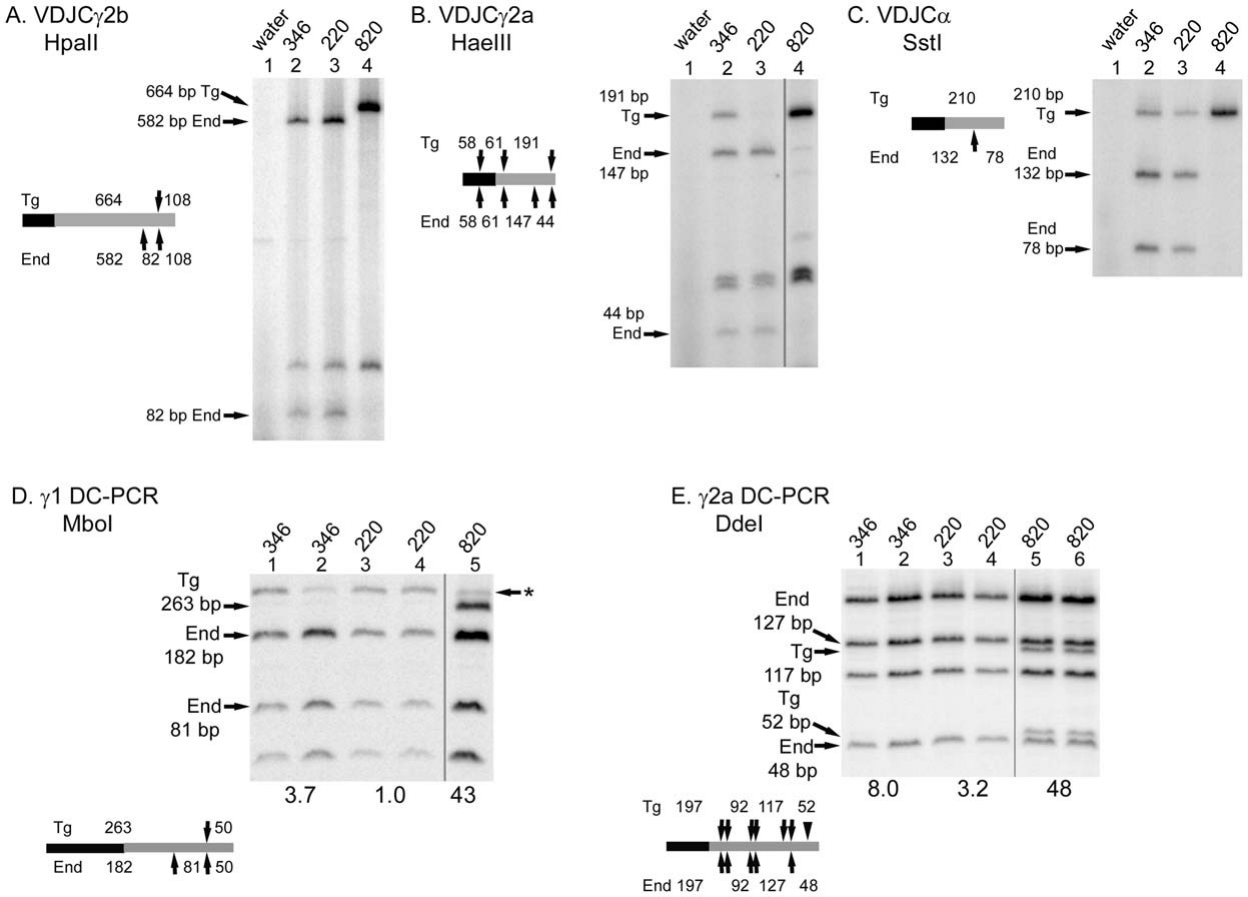
Recombination of the truncated H chain transgenes frequently involves endogenous H chain constant region genes, and is inhibited at the level of transgenic DNA. VDJCγ2b (A), VDJCγ2a (B) and VDJCα (C) were amplified by RT-PCR. The products were digested with restriction enzymes that distinguish the transgene CH region from the endogenous CH region (grey rectangles in the schematic). The VDJ sequences are depicted as black rectangles in the schematics. (D) γ1 DC-PCR products were amplified from DNA of transgenic B cells cultured in LPS+IL-4. The DC-PCR products were digested with *Mbo*I, distinguishing the transgenic and endogenous products, as illustrated in the schematic (black, µ part of the product; grey, γ1 part of the product; arrows, restriction sites). The asterisk indicates a non-specific PCR product. The average ratio (of two or three experiments, multiplied times 100) of the signal in the Tg fragments relative to the signal in the End fragments is shown below transgenic line. Results from individual experiments: line #346, ratios (x 100) of 0, 0, and 11; line #220, ratios of 0, 0.3, and 7.3; line #820, ratios of 23 and 63. (E) γ2a DC-PCR products were amplified from DNA of transgenic B cells cultured in CD40L+interferon-γ, and digested with *Dde*I. Presentation as Part D. The black triangle in the schematic represents a 4-bp insertion in the transgene relative to the endogenous genes. Results from individual experiments: line #346, ratios of 5, 4, and 15; line #220, ratios of 3, 4, and 2.7; line #820, ratios of 42, 44 and 59; nontransgenic mouse, a ratio of 6.9 (one experiment).

In virtually every case where CSR of the transgene is reduced by mutation of regulatory elements, we have detected mRNA with the transgenic VDJ attached to an endogenous CH region [18], [21]. This phenomenon, which is most easily explained as “trans-recombination”, was discovered by Selsing and colleagues [31] and the mechanism has been thoroughly studied by them and by Manser and colleagues [32], [33]. We put aside the interesting biological and mechanistic aspects of this recombination event, focusing on the fact that potential trans-recombination with the endogenous locus can lead to an overestimation of CSR on the transgene, as detected by the amount of VDJCH transcripts. We amplified VDJCH transcripts from various RNA samples and digested the products with restriction enzymes that distinguish the transgenic and endogenous CH genes, as illustrated in the schematics in Fig. 6A, B, C. In virtually all of the transcripts from the γ2b gene (Fig. 6A) and the γ2a gene in line #220 (Fig. 6B), the transgenic VDJ is attached to the endogenous CH gene. The majority of transcripts from the γ2a gene in line #346 (Fig. 6B) and the α genes (Fig. 6C) attach the endogenous CH to the transgenic VDJ. To examine the most extreme example, VDJCγ2b transcripts from line #346 are less abundant than a 1∶25 dilution (4%) of cDNA from a wild type H chain transgene (Fig. 4A). However, at least one-half of those transcripts are derived from the endogenous constant region (Fig. 5B, lane 2). Therefore, at most, CSR to Cγ2b on the truncated line #346 H chain is 2% of wild type. Similar analyses for other H chain genes from #346 and from #220 result in even lower values of CSR. CSR of the truncated H chain loci is highly disabled for all H chain genes in both line #346 and line #220.

### Switch Recombination in DNA

Although the reduction in transgenic µ expression is often not statistically significant (Figs. 1, 3, and 4), it may indicate that the deletion of some of the 3′ enhancers and the HS5-7 region may reduce transcription of the H chain genes post switch recombination. It is possible that CSR occurs at near wild type levels on the transgene, but that the resulting switched γ and α genes cannot be transcribed well [34], [35], and do not result in wild type levels of mRNA (Figs. 4 and 5) or secreted IgG (Fig. 3). DC-PCR, developed by Chu and colleagues, allows one to measure CSR at the level of DNA recombination, regardless of how well those switched H chain genes are subsequently expressed [29]. For our transgenic studies, we added restriction enzyme digestion of the DC-PCR product to distinguish CSR on the transgene from that on the endogenous genes (Fig. 6DE). Fragments indicating CSR to γ1 (Fig. 6D) or to γ2a (Fig. 6E) on an intact H chain transgene (line #820 with one transgenic copy) were easily detected, at approximately the one-half the intensity of fragments indicating CSR of the two endogenous genes. The results from lines #346 and #220 were entirely consistent with a low level of CSR on the truncated transgenes. Averaging the data from the three samples we tested from each truncated transgene, the transgene to endogenous ratio (x 100) ranged from 1 to 8, similar to the background level in nontransgenic C57BL/6 DNA, but reduced relative to the average ratios of 43 and 48 for wild type transgenes (Fig. 6DE). It is possible that truncation of the H chain locus results in poor post switch enhancement of transcription, and this transcriptional deficit contributes to poor H chain expression [34]. Nevertheless, CSR at the level of DNA recombination is dramatically impaired.

## Discussion

Class switch recombination was dramatically reduced for α and all four γ genes in transgenes lacking either the insulator region and HS4, or the insulator region, HS4, HS3B, and HS1,2. For most H genes, CSR was reduced to 1–5% of the levels of CSR from wild type transgenes (Figs. 3, 4, 5, 6). For example, transgenic VDJCγ1 expression in lines #346 and #220 was indistinguishable from the background level of nontransgenic B cells (Fig. 4C). Average secretion levels of 101 or 131 µg/ml of transgenic IgG1^a^ from truncated transgenes compared to 12 µg/ml background levels in nontransgenic mice might suggest some small amount of CSR (Fig. 3A). However, in both the assay for secreted IgG1 and the assay for expression of VDJCγ1 mRNA, the difference in transgenic γ1 expression from truncated H chain transgenes was not statistically different from background levels from nontransgenic B cells. With enough replication of careful experiments, one could probably determine if there were some level of CSR remaining from the truncated H chain transgenes. We focused on the fact that CSR is severely disabled in both line #346 and line #220 transgenes, to less than 2% of wild type levels, and did not attempt to exactly quantify the magnitude of the impairment.

Expression of the transgenic VDJ sequences with endogenous Cγ or Cα sequences (Fig. 6ABC) complicated the quantification of the amount of CSR by the truncated transgenes. Mechanistic aspects of these putative “trans-recombination” events are of interest. Durdik, Guisti, and colleagues were able to detect products of trans-recombination after repeated immunization in vivo [31], [33], but the rate of trans-recombination in light of strong selection by antigen was difficult to estimate. We would suggest that the rate of trans-recombination to the endogenous γ2a gene is at most 1% of the rate of CSR by a wild type transgene, because the efficiency of VDJCγ2a expression from the truncated transgenes is about 1% of that from the wild type transgene (Fig. 5B), and trans-recombination can be about equal to CSR by the truncated transgene (Fig. 6B, line #346). The mechanism of the trans-recombination is also an interesting subject for future studies. Even though some recombination events take place in switch regions [32], others can take place even if donor transgenes lack switch regions [33]. Even though the majority of the recombination events require AID, a portion of the trans-recombination events may be independent of AID [36]. It seems likely that the final products of trans-recombination arise from multiple recombination pathways [32], [33], [36].

The truncation of the H chain transgenes had a more variable effect on germline transcription. Germline transcription of some H chain genes was not altered (γ2b, Fig. 4D), whereas germline transcription of other H chain genes was greatly reduced (γ3 and α, Fig. 4B). Germline transcription of the γ1 and γ2a genes in line #346, with the larger truncation, was not substantially different from germline transcription of these two γ genes in intact H chain transgenes (Fig 4C and 4E). On the other hand, germline transcription of the γ1 and γ2a genes in line #220, with the truncation of HS4 and sequences more 3′, was dramatically reduced. One interpretation of these results would emphasize more the differences between line #346 and line #220 in the truncation of H chain transgenic sequences. Sequences between HS3A and HS4 may inhibit germline transcription of the γ1 and γ2a genes in the line #220 trangene, but since these sequences are deleted in line #346, germline transcription of these two H chain genes would precede at higher levels (Fig. 4C and 4E). An alternative interpretation would emphasize the difference in chromosomal insertion site for these two H chain transgenes. Insertion site effects in line #346, resulting from positive control elements in sequences that flank the transgene, would increase the germline transcription of the γ1 and γ2a genes. A different version of this alternative interpretation is that negative control elements in the sequences that flank the transgene in line #220 inhibit expression of γ1 and γ2a germline transcripts.

Nevertheless, the functional differences between the transgenes in line #346 and in line #220 are minor compared to the deficiency they share–the nearly complete lack of CSR. The lack of CSR may result from negative elements that inhibit CSR in the sequences flanking the insertion site in both transgenic lines. Supporting the notion of an insertion site effect, the region deleted by both truncations includes elements with the structure and activities of an insulator element [22], [23]. Loss of this insulator region may make CSR activities sensitive to the transgene insertion site. It is important to note that the transgenic µ gene is expressed well in lines #346 and #220 (Figs. 1B and 4A). Hence, any potential insertion site effects do not alter transgene expression in general, but rather CSR and some aspects of germline transcription. A second interpretation for the lack of CSR in both transgenic lines is deletion of enhancers for CSR. In this regard, since the phenotypes of transgene germline transcription and CSR are more similar for lines #346 and #220 than they are different, it may be useful to consider that both HS4 and HS5-7 are deleted by the two truncations. In the endogenous locus, there is no evidence that either HS4 [13] or HS5-7 [25] include an enhancer element that is required for CSR or germline transcription. Experiments investigating enhancer activity 3′ of HS4 also argue against enhancer elements in this region that are strictly required for CSR [37]. Therefore, if deletion of enhancer elements is the cause of the poor CSR in lines #346 and #220, one might surmise that at least two redundant elements, one in HS4 and a second in HS5-7 or in the sequences more 3′ were deleted as a result of the truncation. The hypothesis that two redundant regulatory elements are deleted in the truncation H chain transgenes has precedent in other studies of regulatory elements in the 3′ regulatory region. Deletion of a single enhancer element does not alter CSR [12]–[14], [25], but deletion of two enhancers often reduces germline transcription and CSR of some H chain genes [17], [20], [21].

## Supporting Information

Table S1 **PCR amplification of cDNA for the detection of germline and post-switch transcripts.** In general, the same CH primers were used for VDJ, Iμ, and germline transcripts. A different primer was used for γ2b germline transcripts, in order to include a polymorphic *Hpa*II site in the RT-PCR product. γ2a germline transcripts were distinguished by a migration polymorphism between the transgene and endogenous genes.
(DOCX)

Table S2 **DC-PCR analysis of CSR in DNA.**
(DOCX)

Table S3 **Analysis of transgene content.**
(DOCX)
