# Supplementary material for: Transgenes of the Mouse Immunoglobulin Heavy Chain Locus, Lacking Distal Elements in the 3′ Regulatory Region, Are Impaired for Class Switch Recombination
Source: PLoS One. 2013 Feb 8;8(2):e55842. doi: 10.1371/journal.pone.0055842 (PMC3568100; doi:10.1371/journal.pone.0055842)
Supplement: Table S1 — PCR amplification of cDNA for the detection of germline and post-switch transcripts. In general, the same CH primers were used for VDJ, Iμ, and germline transcripts. A different primer was used for γ2b germline transcripts, in order to include a polymorphic HpaII site in the RT-PCR product. γ2a germline transcripts were distinguished by a migration polymorphism between the transgene and endogenous genes. (DOCX) [file pone.0055842.s001.docx]

| cDNA | upstream primer | downstream primer | Annealing, cycles | Restriction digest | Fragments (bp) |
| --- | --- | --- | --- | --- | --- |
| VDJ CH | GTCTATTTCTGTGCAAGATCGAAT |  | 69^o^C,  32 cycles | none | 210-644 bp |
| Iμ | CACCCATCCACCTGGCTGCTCA |  | 65^o^C,  32 cycles | none | 226-660 bp |
| Cμ |  | AATGGTGCTGGGCAGGAAGT (CH1) | 65^o^C,  30 cycles | none | 199 bp |
| Iγ3 | TGATGGGATATATCAGGATACC | TACTGGGCTTGGGTATTCTAG (γ3 hinge) | 61^o^C,  30 cycles | *Pst*I | Transgenic: 127+69 Endogenous:196 Both: 145+121 |
| Iγ1 | GACGGCTGCTTTCACAGCTT | GCATGATGGGAAGTTCACTGACTG (CH2) | 58^o^C,  30 cycles | *Mbo*I | Transgenic: 217+100 Endogenous:317 Both: 261 |
| Iγ2b | gagcactgggcctttccagaacta | AGATGGTTCTCTCGATGGGTGA (CH2, germline transcripts) | 61^o^C,  30 cycles | *Hpa*II | Transgenic: 761 Endogenous: 691+82 Both: 108 |
| post-switch γ2b transcripts |  | GGGCATTTGTGACACTCCTTGCA (γ2b hinge,  VDJ and Iμ transcripts) | 69^o^ or 65^o^C,  32 or 30 cycles | none | 440 or 446 bp |
| Iγ2a | GCTGATGTACCTACCTGAGAGAG | GTCCACCTTGGTGCTGCTT (CH1) | 61^o^C,  30 cycles | none | Transgenic: 403 Endogenous:399 Both: 280 (splice variant) |
| α | CCAGGCATGGTTGAGATAGAG (Iα) | CCCAGGTCACATTCATCGTGC (CHI) | 60^o^ C, 35 cycles | *Sst*I and *Pst*I | Transgenic: 202 Endogenous: 123+79 Both: 131 |
| HPRT | GCTGGTGAAAAGGACCTCT | cacaggactagaacacctgc | 54^o^C, 25 cycles | none | 249 bp |
