# Supplementary material for: Transgenes of the Mouse Immunoglobulin Heavy Chain Locus, Lacking Distal Elements in the 3′ Regulatory Region, Are Impaired for Class Switch Recombination
Source: PLoS One. 2013 Feb 8;8(2):e55842. doi: 10.1371/journal.pone.0055842 (PMC3568100; doi:10.1371/journal.pone.0055842)
Supplement: Table S2 — DC-PCR analysis of CSR in DNA. (DOCX) [file pone.0055842.s002.docx]

| Gene | Primer near μ gene | Primer near γ gene | Annealing | Digestion | Fragments |
| --- | --- | --- | --- | --- | --- |
| γ1 | GGAGACCAATAATCAGAGGGAAG (5’ Sμ) | GGTCCAGTTGAGTGTCTTTAGAG D78344 residues 8781-8803 | 65^o^ C, 35 cycles | *Mbo*I | Transgenic: 263 Endogenous: 182+81 Both: 50 |
| γ2a | GGAGACCAATAATCAGAGGGAAG (5’ Sμ) | GCTCCTGGATGCAGCTAATGC D78344 residues 48615-35 | 65^o^ C, 35 cycles | *Dde*I | Transgenic: 117+52 Endogenous: 127+48 Both:197+92+31+20 |
