# Supplementary material for: Transgenes of the Mouse Immunoglobulin Heavy Chain Locus, Lacking Distal Elements in the 3′ Regulatory Region, Are Impaired for Class Switch Recombination
Source: PLoS One. 2013 Feb 8;8(2):e55842. doi: 10.1371/journal.pone.0055842 (PMC3568100; doi:10.1371/journal.pone.0055842)
Supplement: Table S3 — Analysis of transgene content. (DOCX) [file pone.0055842.s003.docx]

| Gene  segment | upstream primer | downstream primer | Annealing | Restriction digest | Fragment sizes, in bp |
| --- | --- | --- | --- | --- | --- |
| Tg VDJ exon | CACCCAAGTCTATGGCAGTA (90 bp 5’ of VDJ exon) | TCCCTAGTCCTTCATGACCT (90 bp 3’ of JH2) | 57^0^C | none | 800 |
| JH | GGCCAAGGCACCACTCTCAGAG (JH2) | CAGTGACCAGAGTCCCTTGGCC (JH3) | 68^o^ C | *Bam*HI and *Dde*1 | Transgene: 103 Endogenous: 67+36 Both: 128+99+27 |
| Cγ3 | GCATCCTTGTAGGACC AAGGC NT_114985.2 residues 1276124-44 | TACTGGGCTTGGGTATTCTAG NT_114985.2 residues 1276887-67 | 62^o^ C | *Pst*I | Transgene: 129+129 Endogenous: 258 Both: 506 |
| Cγ1 | CTGACTCCTAAGGTCACGTGTG D78344 residues 12993-13014 | GCAGGTCAGACTGACTTTATCC D78344 residues 13452-13431 | 61^o^ C | *Mbo*I | Transgene: 414 Endogenous: 336+78 Both: 47 |
| Cγ2b | GAGGGAGATACTGTCTCTGCCTCC D78344 residues 34322-345 | AGATGGTTCTCTCGATGGGTGA D78344 residues 35014-34993 | 66^o^ C | *Hpa*II | Transgene: 585 Endogenous: 503+82 Both: 108 |
| Iγ2a | ACTAAAGCTGCAGACACATAC D78344 residues 44111-31 | GTATTGGTACTGCAGGAACC D78344 residues 44420-400 | 62^o^ C | *Ase*I | Transgene: 197+112 Endogenous: 309 |
| Cε | GACCTGTCAACATCACTGACCC AJ851868.3 residues 1,576,015-036 | GAGACACATTTAGGATGTGGCC AJ851868.3 residues 1,576,135-113 | 60^o^ C | *Nsi*I | Transgene: 60+61 Endogenous: 121 |
| Cα | ACAGCCAGAACTGTTGGTCAGC AJ851868.3 residues 1,588,525-546 | CCCAGGTCACATTCATCGTGC AJ851868.3 residues 1,588,817-797 | 62^o^ C | *Sac*I | Transgene:293 Endogenous:215+78 |
| HS3A | GCGGCCGCGATACTGGTCACTGGG AF450245 residues 364-379 with an irrelevant *Not*I site added to the 5’ end | CCCAGAACTCAGTGCTGTAGAC AF450245 residues 1059-1038 | 60^o^ C, 5 cycles 66^o^ C, 30 cycles | *Dra*I | Transgene: 421+312 (with 38 bp loxP insertion)  Endogenous: 695 |
| HS1,2 | GCTGCAGGTTCACCCCAACC AF450245 residues 11938-11957 | GACAAGCAGGGAGGTGACAGGCTG AF450245 residues 12416-12392 | 68^o^ C | *Mbo*I | Transgene: 281 Endogenous: 241+40 Both: 198 |
| HS3B | AGTCCAGAGGACTGTCCTCCAT AF450245 residues 23999-24020 | TGAGGTCAGCCAGCATCACCC AF450245 residues 24209-24189 | 66^o^ C | *Taq*I | Transgene: 142+69 Endogenous: 211 |
| HS4 | GTGCTGTCTCTGCATCCTTTGCC AF450245 residues 27971-93 | GTTTCTGGGTGTCTCTGTGTCTGTTC AF450245 residues 28894-69 | 68^o^ C | *Hin*fI | Transgene: 177 (with 34 bp loxP insertion) Endogenous: 139 Both: 446+284+55 |
| 38.1 | CCTACCCTGTCTGGTTGAGGC AF45245 residues 37789-809 | GTTGGCAAGAGGACTCACTACGTC AF450245residues 38213-190 | 62^o^ C, 32 cycles | *Sty*I | Transgene: 266+158 Endogenous: 424 |
